# High-Dose Tranexamic Acid in Patients Underwent Surgical Repair of Aortic Dissection Might Reduce Postoperative Blood Loss: A Cohort Analysis

**DOI:** 10.3389/fsurg.2022.898579

**Published:** 2022-06-14

**Authors:** Jingfei Guo, Liang Cao, Hongbai Wang, Guangyu Liu, Yong Zhou, Lijing Yang, Yuan Jia, Su Yuan

**Affiliations:** Department of Anesthesiology, Fuwai Hospital, National Center for Cardiovascular Diseases, Chinese Academy of Medical Sciences and Peking Union Medical College, Beijing, China

**Keywords:** tranexamic acid, dosage, aortic dissection, blood loss, transfusion

## Abstract

**Introduction:**

While tranexamic acid (TXA) is widely used in patients with acute type A aortic dissection (ATAAD) who undergo surgical repair to reduce blood loss and transfusion requirement, the optimal dosage of TXA is unknown in these patients.

**Materials and Methods:**

This was a retrospective cohort study that compared high-dose (>50 mg/kg) and low-dose TXA (≤50 mg/kg) in patients with ATAAD who underwent surgical repair. Propensity score matching (PSM) was performed between the two groups and results were analyzed in matched cases. The primary outcome was postoperative blood loss within 3 days after surgery. The secondary outcomes were total blood loss after surgery and perioperative blood transfusion, and safety outcomes were also assessed.

**Results:**

Through medical record screening, 529 patients were identified. After PSM, 196 patients in the high-dose group and 196 patients in the low-dose group were matched and included in the final analysis. Postoperative blood loss in 3 days after surgery was 940 mL (710–1,010 mL) in the low-dose group and 695 mL (620–860 mL) in the high-dose group. The difference was statistically significant (*P* < 0.001). Total postoperative blood loss was also statistically less in the high-dose group compared to the low-dose group (1,890 mL (1,410–2,100 mL) vs. 2,040 mL (1,460–2,320 mL), *P *= 0.032). No difference was found between the two groups in transfusion and safety outcomes.

**Conclusion:**

In ATAAD patients who underwent surgical repair, high-dose TXA significantly reduced postoperative blood loss compared to low-dose TXA, while no difference in transfusion or adverse events was found.

## Introduction

Surgery is the primary treatment for acute type A aortic dissection (ATAAD) (1). These patients frequently have excessive perioperative bleeding and the consequent increased need for allogeneic transfusions. The bleeding is due to extensive surgical dissection, the fragile tissues of the diseased aorta, and changes in coagulation processes related to cardiopulmonary bypass (CPB) and deep hypothermia circulatory arrest. Hemostatic medications have been widely used in ATAAD patients who underwent surgical repair.

Tranexamic acid (TXA) is a synthetic analogue of the amino acid lysine and can block lysinebinding sites on plasminogen, thus preventing its conversion to plasmin and executing antifibrinolytic function (2). The effect of TXA to reduce perioperative blood loss and blood transfusion has been well established in many trials and its usage in cardiac surgeries has been recommended in the guideline (3), but the optimal dosage of TXA in cardiac surgery has always been a problem of debate. The dosage of TXA has not been studied in patients with ATAAD who underwent surgical repair. In this retrospective cohort study, we aim to compare the hemostatic effect and safety outcomes between high-dose and low-dose TXA groups in these patients.

## Materials and Methods

### Patient Population and Propensity Score Matching

All study procedures have been conducted according to the principles expressed in the Declaration of Helsinki and its subsequent amendments. The protocol was approved by the Medical Ethics Committee of the Fuwai Hospital. Written informed consent from all patients for this study was waived. This report adheres to Strengthening the Reporting of Observational Studies (STROBE) guidelines.

We screened all patients who underwent surgical correction of ATAAD from January 2011 to December 2015. Only patients who underwent total arch replacement and frozen elephant trunk (TAR + FET) were studied. Patients who had a hybrid procedure, off-pump surgery, or partial aortic arch replacement were excluded. Then we searched for intraoperative TXA usage in these patients and excluded those who did not use TXA. According to TXA dosage, we divided the patients into two groups: the high-dose group (TXA > 50 mg/kg) and the low-dose group (TXA ≤ 50 mg/kg). The cut-off value of 50 mg/kg was set according to our previous study (4). Propensity score matching (PSM) was performed between the two groups. Blood loss, blood transfusion and safety outcomes were analyzed between matched cases after PSM.

### Data Acquisition

We collected demographic characteristics and perioperative data in patients, including age, sex, body mass index, history of smoking, history of diabetes, history of hypertension, history of peripheral vascular disease, history of chronic kidney disease, history of cerebral vascular disease, history of heart failure, preoperative ejection fraction, preoperative hemoglobin level, preoperative platelet count, operation time and CPB time. In total, 15 covariates were included in baseline information.

The primary outcome was postoperative blood loss within 3 days after surgery, intraoperative blood loss was not included. The secondary outcomes were total blood loss after surgery and perioperative blood transfusion, including the transfusion rate and transfusion volume of red blood cells, fresh frozen plasma and platelet. We also incorporated safety outcomes. In-hospital death, 30-day mortality, perioperative myocardial infarction, stroke, pulmonary embolism, deep vein thrombosis, acute kidney injury and seizure were recorded. Other outcomes included postoperative hospital stay, ICU stay and duration of mechanical ventilation.

### Statistical Analysis

The continuous variables were tested for normality. They were expressed as mean ± standard deviation if normally distributed, or expressed as median and quartiles if not normally distributed. The categorical variables were presented as numbers and percentages. For normally distributed continuous variables, student’s-t test was performed to assess the statistical difference between groups. Mann-Whitney U test was performed for continuous variables that weren’t normally distributed. The *χ*^2^ test or Fisher’s exact test was performed, as appropriate, for categorical variables. Statistical differences of baseline information between groups after matching were assessed with the paired student’s t-test for continuous variables in the normal distribution, and Wilcoxon rank test in non-normal distribution, and the paired *χ*^2^ test for categorical variables. Conditional logistic regression was performed for outcome analysis after matching. The odds ratio and 95% confidence interval were calculated for the binary outcomes. Two-sided *P* values of less than 0.05 were considered statistically significant.

Patients in the high-dose TXA and low-dose TXA groups were 1:1 matched according to the propensity score using a caliper width of 0.01, nearest neighbor matching method without replacement. Fifteen covariates were selected for PSM, including age, sex, body mass index, smoke, diabetes, hypertension, peripheral vascular disease, chronic kidney disease, cerebral vascular disease, heart failure, ejection fraction, preoperative hemoglobin level, preoperative platelet count, operation time, and CPB time. All statistical analyses were performed with IBM SPSS Statistics, version 22.0 (IBM Corp., Armonk, NY, USA). A *post hoc* statistical power was calculated to estimate the existing sample size using G*power software, version 3.1 (5), alpha was set at 5%.

A sensitivity analysis was performed by multivariate linear regression. All 600 patients with ATAAD underwent TAR + FET including those who did not use TXA were analyzed. The above 15 variables and TXA dosage were selected as covariates and blood loss within 3 days after surgery was selected as the dependent variable. Regression coefficient and significance were calculated.

We also did *post hoc* analyses to analyze the differences between intraoperative blood loss and reoperation for bleeding between the two dosage groups.

## Results

### Study Population and Propensity Score Matching

**Figure 1:**
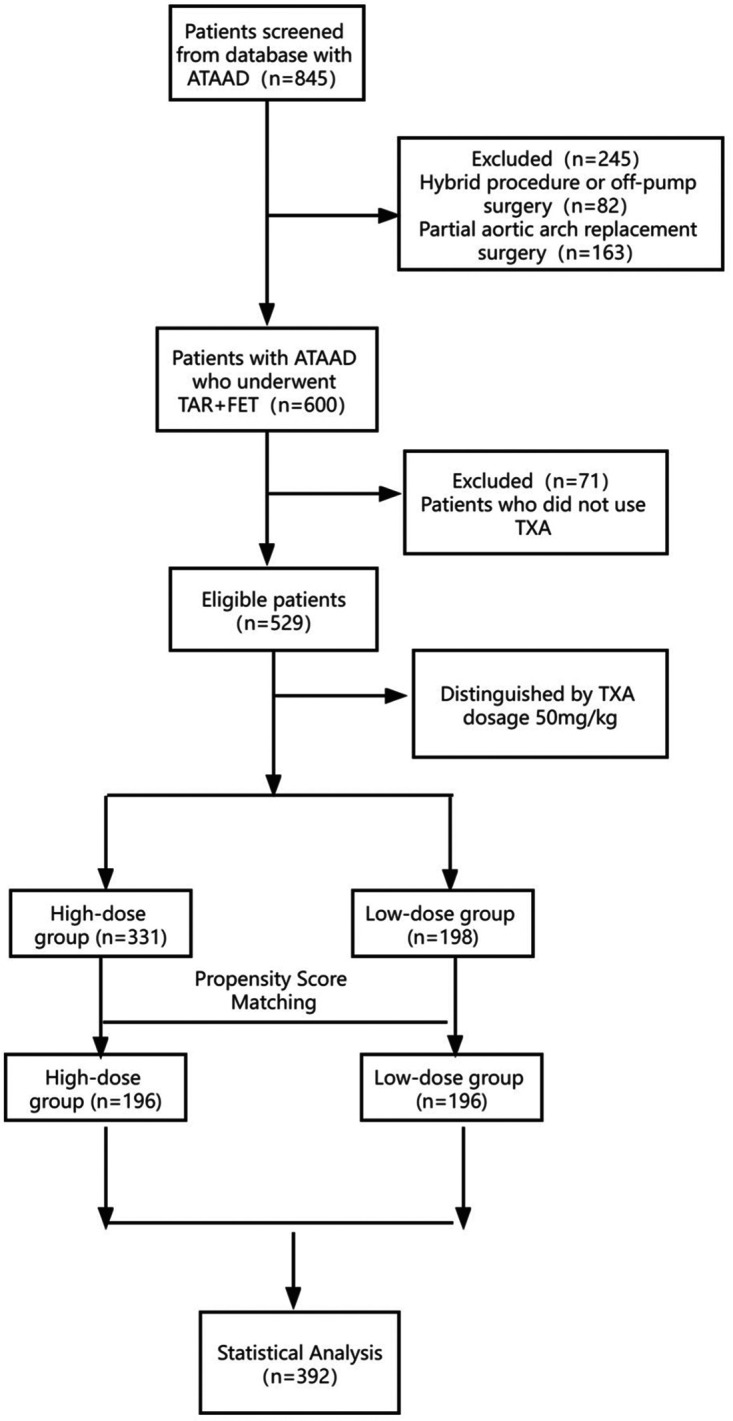
Study flowchart. ATAAD, acute type A aortic dissection; TAR + FET, total arch replacement and frozen elephant trunk; TXA, tranexamic acid.

We identified 845 patients who was diagnosed as ATAAD from January 2011 to December 2015 by database searching. One hundred and sixty-three patients who underwent partial aortic arch replacement surgery and 82 patients who had a hybrid or off-pump surgery were excluded. We thus identified 600 patients with ATAAD who underwent TAR + FET. Seventy-one patients were excluded for not using TXA during surgery. Eventually, 529 patients were included in the analysis (Figure 1). There were 331 patients in the high-dose group (TXA > 50 mg/kg) and 198 patients in the low-dose group (TXA ≤ 50 mg/kg).

**Figure 2:**
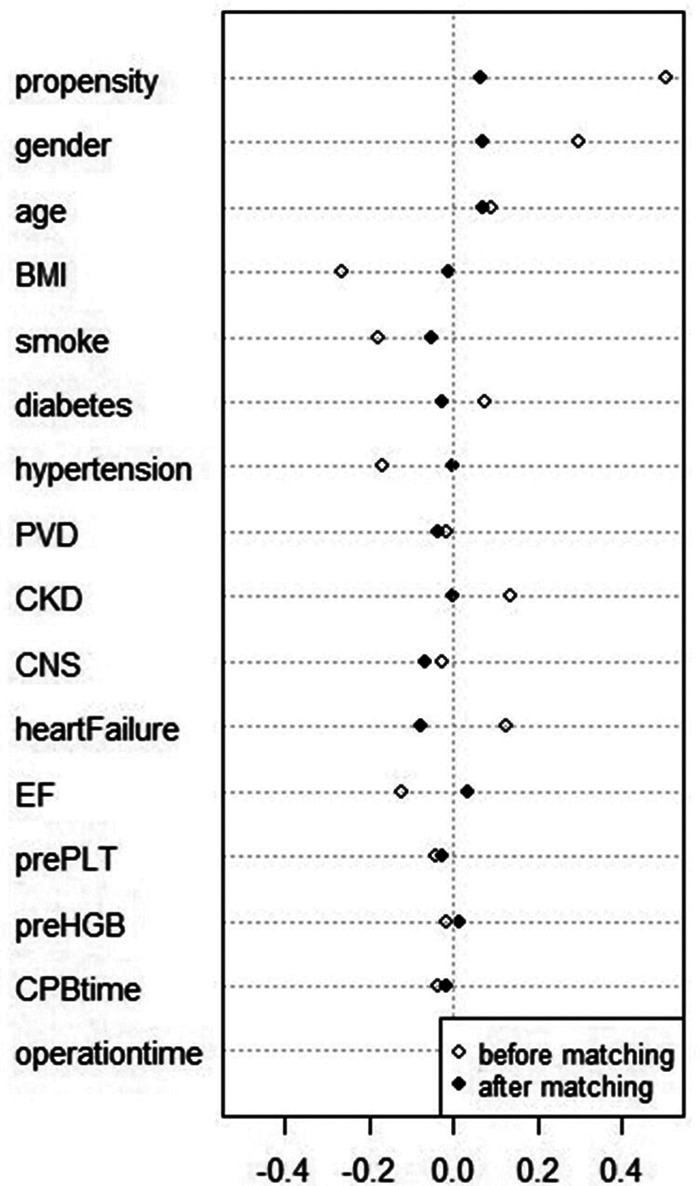
Standard differences of variables before and after matching. BMI, body mass index; PVD, peripheral vascular disease; CKD, chronic kidney disease; CVD, cerebral vascular disease; EF, ejection fraction; prePLT, preoperative platelet count; preHGB, preoperative hemoglobin level; CPB, cardiopulmonary bypass.

After PSM, 196 patients in the high-dose group and 196 patients in the low-dose group were matched based on a standardized difference <0.1 (Figure 2). There were significant differences in several covariates between the high-dose and low-dose groups before matching, while no difference remained between the two groups after matching (Table 1). The average TXA dosages in the low-dose and high-dose groups were 37 and 77 mg/kg, respectively.

**Table 1:** Baseline characteristics before and after PSM.

Baseline Characteristic	Before PSM			After PSM		
	Low-dose group (*n* = 198)	High-dose group (*n* = 331)	*P* value	Low-dose group (*n* = 196)	High-dose group (*n* = 196)	*P* value
Age (y)mean ± SD	47.25 ± 9.25	48.1 ± 10.22	0.315	47.32 ± 9.24	46.61 ± 9.51	0.457
Male *n* (%)	170 (85.9)	240 (72.5)	<0.001	168 (85.7)	170 (86.7)	0.769
BMI>24 kg/M^2^ *n* (%)	155 (78.3)	217 (65.6)	0.002	153 (78.1)	143 (73.0)	0.240
Smoke *n* (%)	95 (48.0)	130 (39.3)	0.050	94 (48.0)	92 (46.9)	0.840
Diabetes *n* (%)	4 (2.02)	11 (3.32)	0.433	4 (2.04)	2 (1.02)	0.685
Hypertension *n* (%)	184 (93.0)	289 (87.3)	0.042	182 (92.9)	178 (90.8)	0.461
PVD *n* (%)	4 (2.02)	6 (1.81)	0.865	4 (2.04)	3 (1.53)	0.703
CKD *n* (%)	7 (3.53)	23 (6.94)	0.100	7 (3.58)	10 (5.10)	0.457
CVD *n* (%)	12 (6.06)	18 (5.44)	0.691	12 (6.12)	13 (6.63)	0.836
Heart failure *n* (%)	3 (1.52)	13 (3.93)	0.188	3 (1.53)	1 (0.51)	0.623
EF% (IQR)	60 (58–62)	60 (58–61)	0.387	60 (58–62)	60 (60–62)	0.391
PreHGB (IQR)	133 (119–146)	133 (118–144)	0.600	133 (119–146)	135 (123–144)	0.757
PrePLT (IQR)	171 (129–222)	170 (138–217)	0.849	171 (129–220)	171 (141–224)	0.528
Operation time (IQR)	390 (335–449)	400 (338–462)	0.430	407 (343–470)	391 (335–451)	0.458
CPB time (IQR)	190 (159–222)	188 (156–221)	0.430	194 (161–232)	188 (156–221)	0.756

*PSM, propensity score matching; BMI, body mass index; PVD, peripheral vascular disease; CKD, chronic kidney disease; CVD, cerebral vascular disease; EF, ejection fraction; prePLT, preoperative platelet count; preHGB, preoperative hemoglobin level; CPB, cardiopulmonary bypass.*

### Primary and Secondary Outcomes

**Figure 3:**
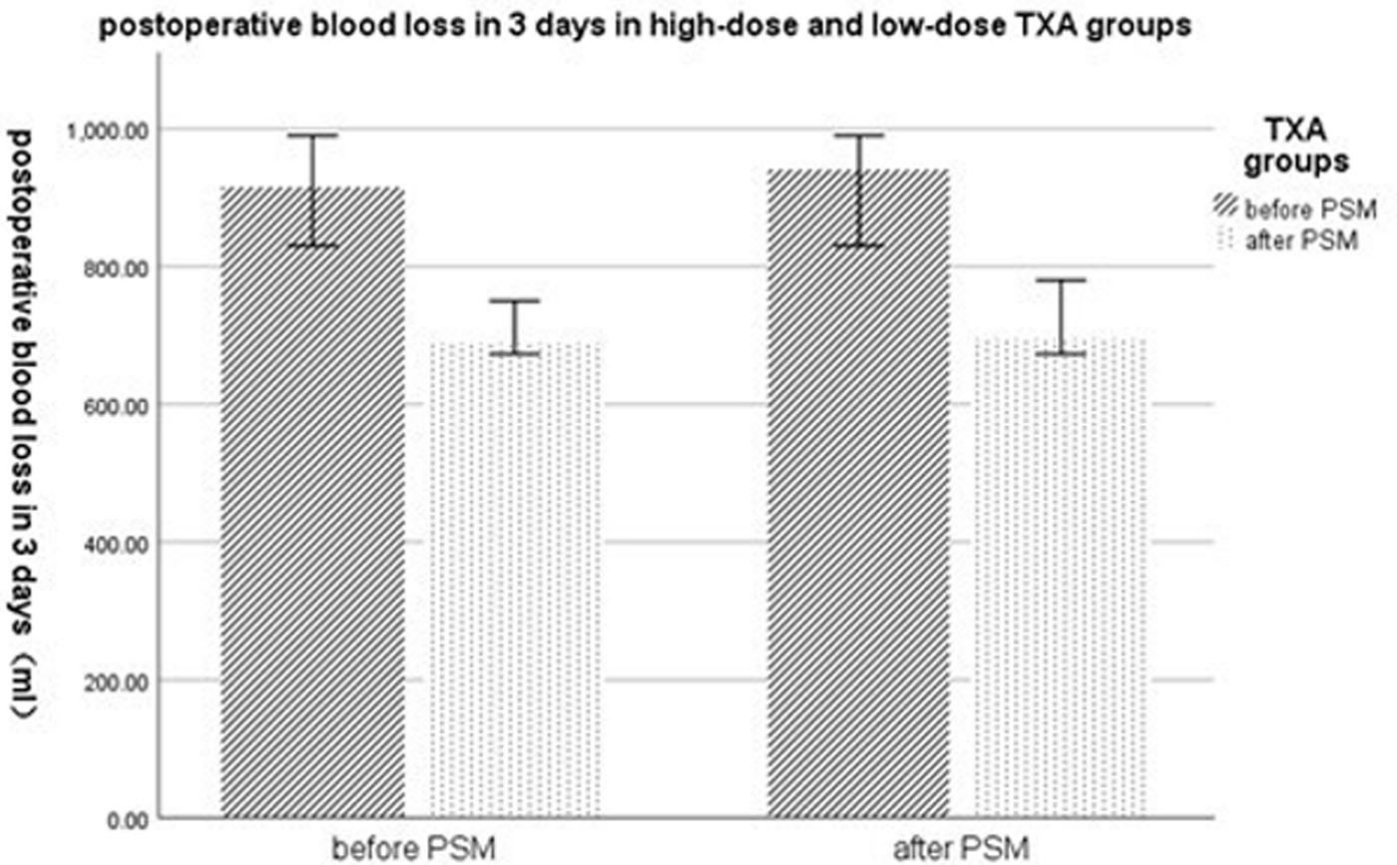
Postoperative blood loss within 3 days in low-dose and high-dose TXA groups. TXA, tranexamic acid; PSM, propensity score matching.

Postoperative blood loss within 3 days after surgery was 940 mL (710–1,010 mL) in the low-dose group and 695 mL (620–860 mL) in the high-dose group. The difference was statistically significant (*P* < 0.001) (statistical power 0.99) (Figure 3). Total postoperative blood loss was also statistically less in the high-dose group compared to the low-dose group (1,890 mL (1,410–2,100 mL) vs. 2,040 mL (1460–2,320 mL), *P *= 0.032) (Table 2). The high-dose and low-dose groups showed no difference in transfusion rate and transfusion volume of any blood products.

**Table 2:** Outcomes for low-dose and high-dose TXA groups after matching.

Outcome	Low-dose TXA group (*n* = 196)	High-dose TXA group (*n* = 196)	OR (95% CI)	*P* value
Blood loss and blood transfusion				
Blood loss in 3 days after surgery /mL (IQR)	940 (710–1010)	695 (620–860)	/	<0.001
Total postoperative blood loss/mL (IQR)	2040 (1460–2320)	1890 (1410–2100)	/	0.032
Total RBC transfusion rate	142 (72.4%)	149 (76.0%)	1.206 (0.766,1.897)	0.419
Total FFP transfusion rate	146 (74.5%)	144 (73.5%)	0.948 (0.604,1.498)	0.818
Total PLT transfusion rate	187 (95.4%)	189 (96.4%)	1.011 (0.393,2.602)	0.982
Total RBC transfusion volume/U (IQR)	4 (0–8)	6 (2–8)	/	0.563
Total FFP transfusion volume/mL (IQR)	600 (50–1150)	600 (0–1100)	/	0.256
Total PLT transfusion volume/U (IQR)	2 (2–3)	2 (1–2)	/	0.069
Adverse Events				
Hospital death	20 (10.2)	17 (8.67)	0.836 (0.424,1.648)	0.604
Death in 30-days after surgery	20 (10.2)	17 (8.67)	0.836 (0.424,1.648)	0.604
Myocardial infarction	2 (1.02)	1 (0.51)	0.497 (0.045,5.531)	1.000
Stroke	8 (4.08)	6 (3.06)	0.742 (0.253,2.180)	0.586
Pulmonary embolism	0	0	/	/
Deep vein thrombosis	2 (1.02)	1 (0.51)	0.497 (0.045,5.531)	1.000
Acute kidney injury	76 (38.8)	75 (38.3)	0.979 (0.652,1.470)	0.917
Seizure	0	1 (0.51)	1.005 (0.995,1.015)	1.000
Other outcomes				
Total hospital stay/d (IQR)	14 (10–18.75)	12 (10–16)	/	0.195
ICU stay/d (IQR)	50.5 (32.25–86.75)	58 (36–93)	/	0.187
Ventilation time/h (IQR)	16 (13–25.5)	17 (13–31)	/	0.340

*TXA, tranexamic acid; RBC, red blood cell; FFP, fresh frozen plasma; PLT, platelet; ICU, intensive care unit.*

In sensitivity analysis, multivariate linear regression found that TXA dosage was negatively correlated with postoperative blood loss within 3 days after surgery (*P* < 0.001) (Supplementary Table S1).

### Safety Outcomes

We found no difference concerning adverse events between the two dosage groups. No significant difference was found in in-hospital death, postoperative 30-day mortality, and the incidence of perioperative myocardial infarction, stroke, pulmonary embolism, deep vein thrombosis, acute kidney injury and seizure between the two groups. The two groups had no difference in in-hospital stay, ICU stay and duration of mechanical ventilation (Table 2).

### *Post hoc* Analyses

For *post hoc* analyses, we found that intraoperative blood loss in the low-dose group was 700(600, 1,200) mL, and intraoperative blood loss in the high-dose group was 600(600, 1,200) mL (median, interquartile range). The difference was not statistically significant (*P* = 0.383). Eight patients in the low-dose group and 5 patients in the high-dose group underwent reoperation because of bleeding. The reoperation rate was 4.1% in the low-dose group and 2.6% in the high-dose group. The difference was also not statistically significant (*P* = 0.397).

## Discussion

Our study demonstrated that high-dose TXA was more effective in reducing postoperative blood loss but not transfusion requirements compared to low-dose TXA in patients with ATAAD who underwent TAR + FET. High-dose TXA did not increase the incidence of thromboembolic events and seizures. To our knowledge, there was no other studies that investigated different dosage regimens of TXA in patients underwent aortic dissection surgery.

TXA has long been used as a hemostatic medication in cardiac surgeries, and it shows the effectiveness in reducing perioperative blood loss and transfusion requirements in both high- and low-dose regimens compared to blank controls (6–8). But the optimal dosage of TXA has always been a problem of debate. While some argue that high-dose TXA was more effective, others believed that lower dosage had the same effect but was safer. Previous studies showed that for overall patients who had cardiac surgery under cardiopulmonary bypass, high-dose TXA might decrease perioperative blood loss compared to low-dose TXA. In an RCT recruiting 160 patients, postoperative blood loss was significantly less in the high-dose group compared to the low-dose group (9). Sigaut et al. observed lower blood loss and less re-exploration surgeries in high-dose group compared to low-dose group (10). Some studies supported that low-dose TXA was equally effective in reducing bleeding. A prospective trial including 1,182 patients studied three different dosage regimens of TXA and found no significant difference in blood loss among the three groups (11). Faraoni et al. did an RCT and no difference was observed between the high-dose and low-dose group in bleeding (12). A less significant difference was discovered between the high-dose and low-dose groups when moving from blood loss to transfusion requirements. Only the RCT conducted by Sigaut et al. reported fewer transfusion requirements in the high-dose group than the low-dose group (10). What was worth noticing was that they recruited patients with high risk of bleeding, which was mostly excluded in other clinical trials. Their inclusion criteria were patients receiving a dual antiplatelet at any time within 5 days of surgery or in the following cases: repeat surgery, surgery of the aorta, intracardiac tumor ablation and surgery for endocarditis. This difference in recruitment could generate a discrepancy in results. Patients with a high risk of bleeding may benefit more from high-dose TXA. Our study targeted at ATAAD patients underwent TAR + FET, these patients had extensive bleeding and transfusion perioperatively. We also found TXA was useful to reduce postoperative bleeding, however, we did not find the difference in blood products transfusion between high- and low-dose groups.

To further clarify the issue of TXA dosage, researchers tried to investigate TXA usage in different types of cardiac surgeries. In a retrospective cohort study that included 42,010 patients who underwent CABG, the high-dose TXA significantly reduced blood loss after cardiac surgery compared to the low-dose TXA (13). While an RCT that enrolled 250 patients found no difference in blood loss between the high-dose and low-dose group (14). Both studies demonstrated no difference in transfusion requirements between the high-dose and low-dose groups for CABG patients. Wang et al. studied 19,687 off-pump CABG patients in a retrospective cohort, there were no difference in either blood loss or blood transfusion between high- and low-dose TXA groups (15). Du et al. randomized 175 patients with cardiac valve surgery and found no statistical difference between the high- and low-dose groups in both postoperative bleeding and the amount and frequency of allogeneic blood transfusion (16).

Several previous studies have shown that for patients with aortic dissection and aortic aneurysm, TXA effectively reduced blood loss and transfusion requirements (17–19). No previous trials compared high and low-dose TXA administration in patients with aortic dissection who underwent surgical repair. Our study showed that blood loss, not blood transfusion, was decreased in the high-dose group compared to the low-dose group. In acute aortic dissection, a large number of thrombi are rapidly formed in the false lumen immediately after onset, which activates the fibrinolytic system (18). Surgical factors also contribute to the activation of fibrinolytic system, such as aortic cross clamp (20), cardiopulmonary bypass (21), and hypothermia (22). All the above factors prompt the need for antifibrinolytic drugs in aortic dissection patients who underwent surgical repair. Due to massive fibrinolytic activity, these patients will theoretically benefit more from high-dose TXA than low-dose TXA. Our research found that high-dose TXA did reduce blood loss more effectively in these patients, but the difference in blood loss may not be as large as to generate a significant difference in blood transfusion according to the current practice. The overall difference in blood loss between the two dosage groups was 150 mL, and this was less than one unit of red blood cells. Even no statistically significant results were found in blood transfusion, a difference of nearly 200 mL in blood loss between high-dose and low-dose TXA groups was still worth noticing. In our institution, RBC transfusion protocol was as follow: (1) hemoglobin below 70 g/L during CPB; (2) hemoglobin below 90 g/L after transfusion of leftover circuit and Cell-saver blood during post-bypass period. This blood management protocol might partly explain our findings.

Although high-dose TXA proved to be more effective in reducing blood loss in patients with aortic dissection who underwent surgical repair, the safety of high-dose TXA administration should be taken into consideration. Whether TXA usage would generate thromboembolic events has always been a problem of concern. There were sporadic reports of a possible relationship between TXA usage and myocardial infarction and pulmonary embolism (23, 24). However, a recent RCT which included 4,662 CABG patients demonstrated that high-dose TXA was not associated with thrombotic complications (6).

Patients with aortic dissection are especially prone to thrombogenesis, so attention should be paid when using high-dose TXA. In our study, there was no difference between the two dosage groups in the incidence of thromboembolic events including myocardial infarction, pulmonary embolism, deep vein thrombosis and stroke. In terms of seizure, several trials identified TXA as the cause for postoperative convulsions, especially with higher doses (≥100 mg/kg total cumulative dose) (25–27). However, no significant difference was found in seizure incidence between two-dosage groups in our study. In all 392 patients, there was only one patient in the high-dose group who used 121 mg/kg of TXA who had a seizure attack. The average dose of TXA used in the high-dose group was 77 mg/kg in our study, and this relatively low dose of TXA may lead to a low incidence of seizure. Our study had limited patient number to study the safety outcomes.

This study had several other limitations. This was a retrospective cohort study. Although a PSM was chosen to simulate an RCT, potential confounding covariates may not be included. Perioperative information was retrospectively extracted from medical records, which may not be as accurate as prospective recordings. RCTs with large sample size are required to further explore this issue.

## Data Availability

The original contributions presented in the study are included in the article/Supplementary Material, further inquiries can be directed to the corresponding author.

## References

[1] Erbel R Aboyans V Boileau C Bossone E Bartolomeo RD Eggebrecht H 2014 ESC guidelines on the diagnosis and treatment of aortic diseases: document covering acute and chronic aortic diseases of the thoracic and abdominal aorta of the adult. the Task Force for the Diagnosis and Treatment of Aortic Diseases of the European Society of Cardiology (ESC). Eur Heart J. (2014) 35:2873–926. 10.1093/eurheartj/ehu281 25173340

[2] Martin J Cheng D. Tranexamic acid for routine use in off-pump coronary artery bypass surgery: evidence base “fait accompli” or more research needed? Anesth Analg. (2012) 115:227–30. 10.1213/ANE.0b013e31825b6746 22826521

[3] Boer C Meesters MI Milojevic M Benedetto U Bolliger D von Heymann C 2017 EACTS/EACTA guidelines on patient blood management for adult cardiac surgery. J Cardiothorac Vasc Anesth. (2018) 32:88–120. 10.1053/j.jvca.2017.06.026 29029990

[4] Guo J Gao X Ma Y Lv H Hu W Zhang S Different dose regimes and administration methods of tranexamic acid in cardiac surgery: a meta-analysis of randomized trials. BMC Anesth. (2019) 19:129. 10.1186/s12871-019-0772-0 PMC6631782 31307381

[5] Faul F Erdfelder E Lang AG Buchner A. G*Power 3: a flexible statistical power analysis program for the social, behavioral, and biomedical sciences. Behav Res Methods. (2007) 39:175–91. 10.3758/BF03193146 17695343

[6] Myles PS Smith JA Forbes A Silbert B Jayarajah M Painter T Tranexamic acid in patients undergoing coronary-artery surgery. N Engl J Med. (2017) 376:136–48. 10.1056/NEJMoa1606424 27774838

[7] Shi J Ji H Ren F Wang G Xu M Xue Y Protective effects of tranexamic acid on clopidogrel before coronary artery bypass grafting: a multicenter randomized trial. JAMA Surg. (2013) 148:538–47. 10.1001/jamasurg.2013.1560 23426385

[8] Esfandiari BR Bistgani MM Kabiri M. Low dose tranexamic acid effect on post-coronary artery bypass grafting bleeding. Asian Cardiovasc Thorac Ann. (2013) 21:669–74. 10.1177/0218492312466391 24569324

[9] Jiménez JJ Iribarren JL Brouard M Hernández D Palmero S Jiménez A Safety and effectiveness of two treatment regimes with tranexamic acid to minimize inflammatory response in elective cardiopulmonary bypass patients: a randomized double-blind, dose-dependent, phase IV clinical trial. J Cardiothorac Surg. (2011) 6:138. 10.1186/1749-8090-6-138 21999189 PMC3206427

[10] Sigaut S Tremey B Ouattara A Couturier R Taberlet C Grassin-Delyle S Comparison of two doses of tranexamic acid in adults undergoing cardiac surgery with cardiopulmonary bypass. Anesthesiology. (2014) 120:590–600. 10.1097/ALN.0b013e3182a443e8 23903022

[11] Waldow T Szlapka M Haferkorn M Bürger L Plötze K Matschke K. Prospective clinical trial on dosage optimizing of tranexamic acid in non-emergency cardiac surgery procedures. Clin Hemorheol Microcirc. (2013) 55:457–68. 10.3233/CH-131782 24113504

[12] Faraoni D Cacheux C Van Aelbrouck C Ickx BE Barvais L Levy JH. Effect of two doses of tranexamic acid on fibrinolysis evaluated by thromboelastography during cardiac surgery: a randomised, controlled study. Eur J Anaesthesiol. (2014) 31:491–8. 10.1097/EJA.0000000000000051 24557022

[13] Wang E Yuan X Wang Y Chen W Zhou X Hu S Blood conservation outcomes and safety of tranexamic acid in coronary artery bypass graft surgery. Int J Cardiol. (2022) 348:50–6. 10.1016/j.ijcard.2021.12.017 34920046

[14] Armellin G Vinciguerra A Bonato R Pittarello D Giron GP. Tranexamic acid in primary CABG surgery: high vs low dose. Minerva Anestesiol. (2004) 70:97–107. PMID: 14997082

[15] Wang E Yuan X Wang Y Chen W Zhou X Hu S Tranexamic acid administered during off-pump coronary artery bypass graft surgeries achieves good safety effects and hemostasis. Front Cardiovasc Med. (2022) 9:775760. 10.3389/fcvm.2022.775760 35187119 PMC8854353

[16] Du Y Xu J Wang G Shi J Yang L Shi S Comparison of two tranexamic acid dose regimens in patients undergoing cardiac valve surgery. J Cardiothorac Vasc Anesth. (2014) 28:1233–7. 10.1053/j.jvca.2013.10.006 24447498

[17] Monaco F Nardelli P Pasin L Barucco G Mattioli C Di Tomasso N Tranexamic acid in open aortic aneurysm surgery: a randomised clinical trial. Br J Anaesth. (2020) 124:35–43. 10.1016/j.bja.2019.08.028 31607387

[18] Ahn KT Yamanaka K Iwakura A Hirose K Nakatsuka D Kusuhara T Usefulness of intraoperative continuous infusion of tranexamic acid during emergency surgery for type A acute aortic dissection. Ann Thorac Cardiovasc Surg. (2015) 21:66–71. 10.5761/atcs.oa.13-00339 24583703 PMC4989989

[19] Casati V Sandrelli L Speziali G Calori G Grasso MA Spagnolo S. Hemostatic effects of tranexamic acid in elective thoracic aortic surgery: a prospective, randomized, double-blind, placebo-controlled study. J Thorac Cardiovasc Surg. (2002) 123:1084–91. 10.1067/mtc.2002.120717 12063454

[20] Adam DJ Ludlam CA Ruckley CV Bradbury AW. Coagulation and fibrinolysis in patients undergoing operation for ruptured and nonruptured infrarenal abdominal aortic aneurysms. J Vasc Surg. (1999) 30:641–50. 10.1016/S0741-5214(99)70103-5 10514203

[21] Woodman RC Harker LA. Bleeding complications associated with cardiopulmonary bypass. Blood. (1990) 76:1680–97. 10.1182/blood.V76.9.1680.1680 2224118

[22] Westaby S. Coagulation disturbance in profound hypothermia: the influence of anti-fibrinolytic therapy. Semin Thorac Cardiovasc Surg. (1997) 9:246–56. PMID: 9263343

[23] Gupta PN Mullamalla UR Sabin P Vellappan P. Acute MI in a young hypertensive woman: could it be due to tranexamic acid? BMJ Case Rep. (2013) 2013:bcr2013009979. 10.1136/bcr-2013-009979 PMC3670019 23715846

[24] Taparia M Cordingley FT Leahy MF. Pulmonary embolism associated with tranexamic acid in severe acquired haemophilia. Eur J Haematol. (2002) 68:307–9. 10.1034/j.1600-0609.2002.01607.x 12144537

[25] Manji RA Grocott HP Leake J Ariano RE Manji JS Menkis AH Seizures following cardiac surgery: the impact of tranexamic acid and other risk factors. Can J Anaesth. (2012) 59:6–13. 10.1007/s12630-011-9618-z 22065333

[26] Keyl C Uhl R Beyersdorf F Stampf S Lehane C Wiesenack C High-dose tranexamic acid is related to increased risk of generalized seizures after aortic valve replacement. Eur J Cardiothorac Surg. (2011) 39:e114–21. 10.1016/j.ejcts.2010.12.030 21295991

[27] Murkin JM Falter F Granton J Young B Burt C Chu M. High-dose tranexamic acid is associated with nonischemic clinical seizures in cardiac surgical patients. Anesth Analg. (2010) 110:350–3. 10.1213/ANE.0b013e3181c92b23 19996135

