# Supplementary material for: High-Dose Tranexamic Acid in Patients Underwent Surgical Repair of Aortic Dissection Might Reduce Postoperative Blood Loss: A Cohort Analysis
Source: Front Surg. 2022 Jun 14;9:898579. doi: 10.3389/fsurg.2022.898579 (PMC9237523; doi:10.3389/fsurg.2022.898579)
Supplement: Supplementary file 1 [file Table_1_v1.docx]

|  | | | | | | | | |
| --- | --- | --- | --- | --- | --- | --- | --- | --- |
| Supplemental Table 1. Multivariate Linear Regression for Postoperative Blood Loss Within 3 Days | | | | | | | | |
| **Model** | | | **Unstandardized Coefficients** | |  | **Standardized coefficient** | **T** | **Significance** |
|  |  |  | **B** | **Standard error** |  | **Beta** |  |  |
|  | （constant） | | 763.933 | 419.637 |  |  | 1.820 | .069 |
|  | TXA dosage | | -4.246 | .939 |  | -.200 | -4.520 | **.000** |
|  | Gender | | 1.225 | 71.283 |  | .001 | .017 | .986 |
|  | Age | | .700 | 2.775 |  | .012 | .252 | .801 |
|  | BMI | | -126.778 | 60.017 |  | -.097 | -2.112 | **.035** |
|  | Smoke | | 100.044 | 55.234 |  | .083 | 1.811 | .071 |
|  | Diabetes | | .233 | 154.249 |  | .000 | .002 | .999 |
|  | Hypertension | | 23.496 | 88.278 |  | .012 | .266 | .790 |
|  | PVD | | -189.055 | 187.421 |  | -.043 | -1.009 | .314 |
|  | CKD | | -82.927 | 109.909 |  | -.032 | -.755 | .451 |
|  | CVD | | -25.593 | 109.776 |  | -.010 | -.233 | .816 |
|  | Heart failure | | -14.988 | 172.094 |  | -.004 | -.087 | .931 |
|  | EF | | .062 | 5.197 |  | .001 | .012 | .991 |
|  | PrePLT | | -.572 | 1.398 |  | -.019 | -.409 | .683 |
|  | PreHGB | | -.582 | .334 |  | -.075 | -1.742 | .082 |
|  | CPB time | | .028 | .576 |  | .003 | .048 | .962 |
|  | Operation time | | 1.290 | .372 |  | .223 | 3.466 | **.001** |
|  | |  | | | | | | |

TXA = tranexamic acid, BMI = body mass index, PVD = peripheral vascular disease，CKD = chronic kidney disease，CVD = cerebral vascular disease，EF = ejection fraction，prePLT = preoperative platelet count，preHGB = preoperative hemoglobin level，CPB = cardiopulmonary bypass
